# Fabrication of Nitrogen Based Magnetic Conjugated Microporous Polymer for Efficient Extraction of Neonicotinoids in Water Samples

**DOI:** 10.3390/molecules29102189

**Published:** 2024-05-08

**Authors:** Zhenzhen Xia, Xinghua Teng, Yuqi Cheng, Yujie Huang, Liwen Zheng, Lei Ji, Leilei Wang

**Affiliations:** Shandong Province Key Laboratory of Applied Microbiology, Ecology Institute of Shandong Academy of Sciences, Qilu University of Technology (Shandong Academy of Sciences), Jinan 250014, China; 10431211181@stu.qlu.edu.cn (Z.X.); 10431221193@stu.qlu.edu.cn (X.T.); 15535455369@163.com (Y.C.); huangyj@sdas.org (Y.H.); zhenglw@qlu.edu.cn (L.Z.)

**Keywords:** conjugated microporous polymers, magnetic solid-phase extraction, neonicotinoids, water sample

## Abstract

Facile and sensitive methods for detecting neonicotinoids (NEOs) in aquatic environments are crucial because they are found in extremely low concentrations in complex matrices. Herein, nitrogen-based magnetic conjugated microporous polymers (Fe_3_O_4_@N-CMP) with quaternary ammonium groups were synthesized for efficient magnetic solid-phase extraction (MSPE) of NEOs from tap water, rainwater, and lake water. Fe_3_O_4_@N-CMP possessed a suitable specific surface area, extended π-conjugated system, and numerous cationic groups. These properties endow Fe_3_O_4_@N-CMP with superior extraction efficiency toward NEOs. The excellent adsorption capacity of Fe_3_O_4_@N-CMP toward NEOs was attributed to its π–π stacking, Lewis acid–base, and electrostatic interactions. The proposed MSPE-HPLC-DAD approach based on Fe_3_O_4_@N-CMP exhibited a wide linear range (0.1–200 µg/L), low detection limits (0.3–0.5 µg/L), satisfactory precision, and acceptable reproducibility under optimal conditions. In addition, the established method was effectively utilized for the analysis of NEOs in tap water, rainwater, and lake water. Excellent recoveries of NEOs at three spiked levels were in the range of 70.4 to 122.7%, with RSDs less than 10%. This study provides a reliable pretreatment method for monitoring NEOs in environmental water samples.

## 1. Introduction

Neonicotinoids (NEOs) are a class of synthetic insecticides that have been extensively utilized for pest control [1,2]. Their high efficiency at low doses, broad insecticidal spectrum, and low cost have made them ideal candidates to replace traditional organophosphate and pyrethroid insecticides [3,4]. However, their excessive use has led to their release into the environment [5,6]. NEOs are highly water-soluble, and thus they spread rapidly in natural surface waters. As such, they are inevitably detected in natural waters [7,8]. In addition, NEO residues have been found in food samples [9]. Therefore, NEOs may pose health risks to humans because they bioaccumulate and end up in drinking water and food [10,11]. Many countries and organizations have imposed strict regulations on the amount of NEO residues in food. For example, the Chinese National Standard (GB 2763-2021) [12] specifies the maximum residue limit of seven NEOs in food (cereals, fruits, vegetables, and vegetable oils). However, restrictions on the amount of NEOs in drinking water are rarely listed in Chinese national standards (GB 5749-2022) [13]. Thus, it is necessary to establish an efficient and reliable analytical method to determine the quantity of NEOs in water samples.

Combined with various detection methods, such as ultraviolet (UV) light, diode array detectors (DADs), and mass spectrometry (MS), high-performance liquid chromatography (HPLC) is still used for the quantitative analysis of NEOs [14,15,16]. The direct determination of NEOs is extremely difficult because of their low concentration in complex matrices. Appropriate sample pretreatment methods such as solid-phase extraction (SPE) [14], solid-phase microextraction (SPME) [17], dispersed solid-phase extraction (DSPE) [4,18,19], and magnetic solid-phase extraction (MSPE) [20] are necessary prior to chromatographic analysis. Among those methods, MSPE has generated widespread attention in the sample pretreatment field owing to its inherent advantages, including efficient extraction phenomenon, rapid separation, low consumption of organic solvents, and appropriate recycling of sorbents [21,22]. In recent years, the number of porous materials has increased significantly, and several of them have been coupled with magnetic cores. These materials have been employed as adsorbents for MSPE to facilitate adsorption capacity towards diverse analytes. Magnetic porous materials have also been successfully applied in the extraction of NEOs. These include magnetic graphene oxide (GO) [23], magnetic nanocellulose (MNC) [24], magnetic metal-organic frameworks (MOFs) [18,25], magnetic porphyrin organic polymer [26,27], magnetic hyper-crosslinked polymer (MHCPs) [28], and magnetic covalent organic frameworks [29,30]. However, magnetic graphene oxide, magnetic nanocellulose, and magnetic hyper-crosslinked polymer exhibited a poorly selective extraction capacity towards NEOs, and magnetic MOFs displayed weak stability under acidic and alkaline conditions. A small variety of functional groups was embedded in magnetic porphyrin organic polymers and magnetic covalent organic frameworks. Thus, the development of magnetic porous materials with abundant functional groups and remarkable stability for highly efficient and selective extraction of NEOs is highly desirable.

Conjugated microporous polymers (CMPs), a class of amorphous microporous organic polymers, have low density, extended π-conjugation, large specific surface area, rigid microporous networks, outstanding stability, diverse structural designs, and abundant functional groups [31,32]. These features make CMPs promising candidates as photocatalysts [33], luminescent materials [34], supercapacitors [35], metal ion rechargeable batteries [36], CO_2_ capture and conversion materials [37,38], energy storage [39], fuel cells [40], and flame-retardant materials [41], among others. CMPs exhibit exceptional adsorption performance toward diverse contaminants [42,43]. Because of the introduction of functional groups into the skeleton of CMPs, their adsorption selectivity towards contaminants was remarkably enhanced, which is beneficial in sample pretreatment [44,45]. Ionic CMPs, containing ionic sites in their frameworks, provide efficient and selective adsorption capacity for ionic targets with opposite charges. Considering the polar nature of NEOs, we speculated that introducing quaternary ammonium groups as cationic sites into CMPs could be considered as a prospective platform for the extraction of NEOs, owing to the electrostatic interactions between quaternary ammonium groups and the negative electrostatic potential regions of NEOs. Moreover, the integration of magnetic nanoparticles within CMPs for the fabrication of magnetic CMP as MSPE sorbents has attracted considerable attention [46,47]. However, pretreatment approaches based on magnetic CMPs with quaternary ammonium groups for the detection of NEOs have rarely been reported.

**Scheme 1:**
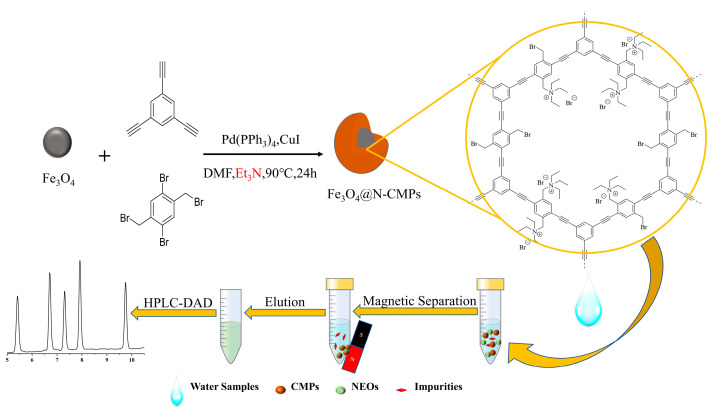
Illustration of the synthesis of Fe_3_O_4_@N-CMP and its MSPE procedure.

Herein, we report the synthesis of a novel nitrogen-based magnetic CMP (Fe_3_O_4_@N-CMP) with quaternary ammonium groups for the efficient MSPE of NEOs in water samples prior to chromatographic analysis (Scheme 1). Fe_3_O_4_@N-CMP was assembled via the Sonogashira–-Hagihara coupling method using Fe_3_O_4_ as the magnetic particle and 1,3,5-triethynylbenzene and 1,4-dibromo-2,5-bis(bromomethyl)benzene as the building units. In the synthesis of Fe_3_O_4_@N-CMP, quaternary ammonium groups were embedded in the CMP skeleton through quaternization between triethylamine and benzyl bromide. This reaction rendered the Fe_3_O_4_@N-CMP positively charged [48]. The infinite π-skeleton, large specific surface area, good chemical stability, and numerous ionic groups endow Fe_3_O_4_@N-CMP an exceptional adsorption capacity toward NEOs. The factors influencing the MSPE performance of Fe_3_O_4_@N-CMP toward the adsorption of NEOs were investigated. A possible adsorption mechanism was discussed. Finally, the developed MSPE approach was combined with HPLC-DAD to measure NEOs in environmental water samples.

## 2. Results and Discussion

### 2.1. Characterization of Fe_3_O_4_@N-CMP

**Figure 1:**
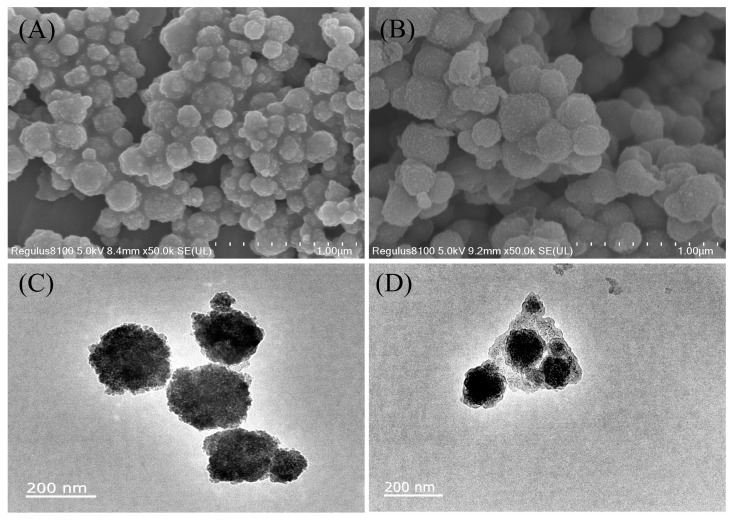
SEM images of Fe_3_O_4_ (**A**), Fe_3_O_4_@N-CMP (**B**); TEM images of Fe_3_O_4_ (**C**), and Fe_3_O_4_@N-CMP (**D**).

The morphologies of the bare Fe_3_O_4_ nanoparticles and Fe_3_O_4_@N-CMP were analyzed by SEM and TEM. As shown in Figure 1A,C, bare Fe_3_O_4_ appeared nearly spherical with a diameter of approximately 200 nm. The Fe_3_O_4_ nanoparticles tended to aggregate. The SEM image (Figure 1B) shows that the surface of Fe_3_O_4_@N-CMP was rough, confirming the formation of a CMP layer. Compared with the TEM image of bare Fe_3_O_4_ (Figure 1C), the TEM image of Fe_3_O_4_@N-CMP (Figure 1D) confirmed that Fe_3_O_4_@N-CMP comprised Fe_3_O_4_ nanoparticles and a CMP layer. Fe_3_O_4_@N-CMP had an irregular lumpy shape, and multiple Fe_3_O_4_ nanoparticles were wrapped in a block of Fe_3_O_4_@N-CMP nanocomposite.

**Figure 2:**
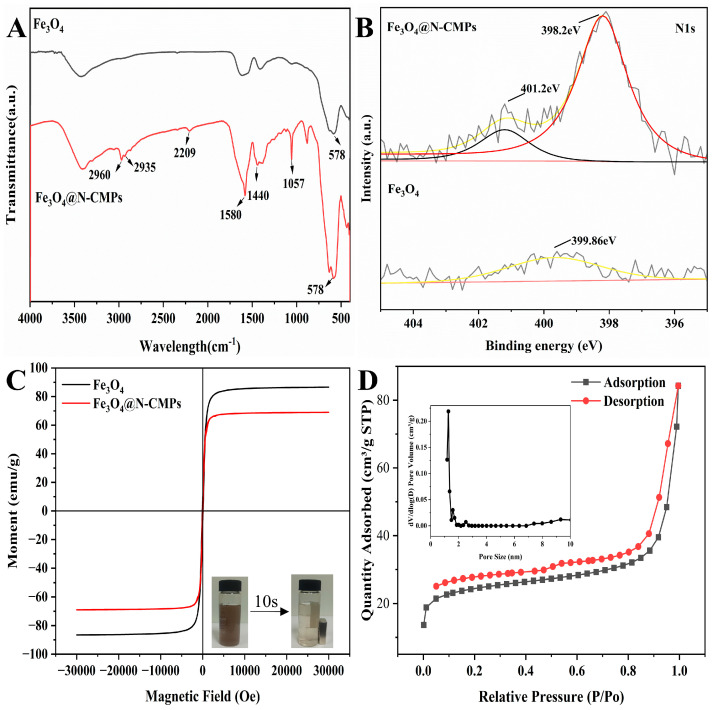
(**A**) FT-IR spectra; (**B**) N 1s XPS spectra; (**C**) magnetization curves (the inset shows magnetic separation of Fe_3_O_4_@N-CMP; (**D**) N_2_ adsorption-desorption isotherm of Fe_3_O_4_@N-CMP (inset: pore size distribution curve).

FT-IR spectroscopy was used to identify the functional groups of Fe_3_O_4_@N-CMP. As indicated in Figure 2A, the characteristic peak at 578 cm^−1^ belonged to the Fe-O-Fe stretching vibration of Fe_3_O_4_, which was present as bare Fe_3_O_4_ and Fe_3_O_4_@N-CMP. The peaks at 1580 and 1440 cm^−1^ were assigned to aromatic C=C stretching vibrations, and that at 2209 cm^−1^ to C≡C stretching vibrations. C–N stretching vibration at 1057 cm^−1^ and aliphatic C-H stretching vibration at 2935–2960 cm^−1^ were observed. This confirmed that the quaternary ammonium salt was successfully loaded on Fe_3_O_4_@N-CMP. XPS analysis was employed to evaluate the elemental compositions of Fe_3_O_4_ and Fe_3_O_4_@N-CMP. As shown in Figure 2B, the spectrum of bare Fe_3_O_4_ hardly shows characteristic N1s peaks. In contrast, the spectrum of the Fe_3_O_4_@N-CMP showed a new N1s peak at 401.2 eV, ascribed to the quaternary ammonium salt [49], demonstrating the existence of a quaternary ammonium group in the Fe_3_O_4_@N-CMP. The content of carbon, hydrogen, and nitrogen in Fe_3_O_4_@N-CMP from element analysis was 20.38%, 1.83%, and 0.89%, respectively. The 50% quaternization percentage of Ph-CH_2_Br groups with Et_3_N was calculated based on the above elemental analysis data of Fe_3_O_4_@N-CMP. The magnetic hysteresis curves in Figure 2C suggest that Fe_3_O_4_@N-CMP possessed superparamagnetic characteristic with the saturated magnetization value of 51.5 emu/g, just a little less than the bare Fe_3_O_4_ (86.3 emu/g). Nevertheless, the separation and recovery of Fe_3_O_4_@N-CMP were easily completed within 10 s using an external magnet. This revealed that Fe_3_O_4_@N-CMP is an appropriate MSPE adsorbent, owing to its satisfactory magnetic separation. The specific surface area and pore size distribution of Fe_3_O_4_@N-CMP were obtained using N_2_ adsorption–desorption isotherms. As shown in Figure 2D, the Brunauer–Emmett–Teller (BET) surface and pore diameter of Fe_3_O_4_@N-CMP are 90.5 m^2^/g and 1.2 nm, respectively, which is in agreement with the microporous features of the bulk CMPs prepared using 1,3,5-triethynylbenzene and 1,4-dibromo-2,5-bis(bromomethyl)benzene. The large external surface area and microporous structure facilitated the adsorption of Fe_3_O_4_@CMP toward contaminants.

### 2.2. Optimization of MSPE Conditions

The parameters that influenced the MSPE performance included the adsorbent amount, extraction time, NaCl concentration, pH, type of elution solvent, and elution time. These parameters were systematically optimized using 30 mL of a NEO-spiked aqueous solution (25 μg/L). Five NEOs were selected to evaluate the adsorption efficiency of Fe_3_O_4_@N-CMP. Each experiment was performed in triplicate.

#### 2.2.1. Effect of Adsorbent Amount

**Figure 3:**
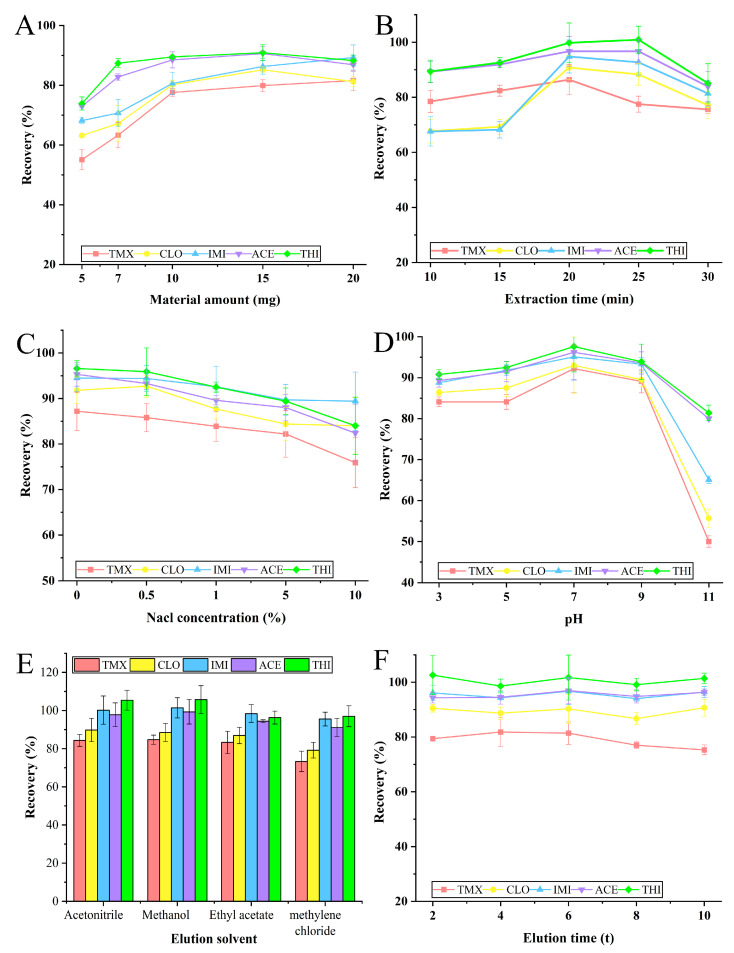
Optimization of MSPE conditions: (**A**) Fe_3_O_4_@N-CMP amount; (**B**) extraction time; (**C**) NaCl concentration; (**D**) pH; (**E**) type of elution solvent; (**F**) elution time (n = 3).

The amount of Fe_3_O_4_@N-CMP is a critical parameter in the MSPE assay. The amount of adsorbent was varied from 5 to 20 mg to study its effect on the extraction of NEOs. As shown in Figure 3A, the recoveries of the five NEOs increased rapidly when the adsorbent amount increased from 5 to 10 mg, but the change was small at higher amounts. Therefore, 10 mg was used in subsequent experiments.

#### 2.2.2. Effect of Extraction Time

Extraction times ranging from 10 to 30 min were used to assess the extraction efficiency of the MSPE procedure. As shown in Figure 3B, the maximum recovery for the five NEOs was obtained at 20 min. Additionally, a remarkable decrease in recovery was observed with longer extraction times. Therefore, 20 min was selected as the optimal extraction time.

#### 2.2.3. Effect of Ionic Strength

Salt addition may be unfavorable for the adsorption of analytes because it increases the viscosity of aqueous solutions. To evaluate the influence of ionic strength towards extraction, the concentration of NaCl in the aqueous solutions was varied in the range of 0% to 10% (*m*/*v*). Figure 3C indicates that the recoveries of the five NEOs decreased with increasing ionic strength. Excessive salinity diminished the transfer of NEOs to the adsorbent material. Therefore, NaCl was not used to optimize the extraction.

#### 2.2.4. Effect of pH

The pH of the sample solution can affect the adsorption efficiency of the NEOs by determining their speciation. The influence of pH was systematically studied by varying the pH from 3 to 11. The pH of the sample solution was adjusted by 1.0 mol/L NaOH or 1.0 mol/L HCl. As shown in Figure 3D, the highest recoveries of the five NEOs were achieved at pH 7. A slight decrease in recovery was observed under acidic conditions, owing to the electrostatic repulsion between the protonated NEOs and Fe_3_O_4_@N-CMP containing quaternary ammonium groups. In alkaline conditions, the ability was attenuated adsorption due to the hydrolysis of the NEOs. Therefore, subsequent experiments were conducted under neutral conditions.

#### 2.2.5. Effect of Elution Solvent

Four organic solvents (methanol, acetonitrile, methylene chloride, and ethyl acetate) were evaluated as eluents. Figure 3E shows that acetonitrile and methanol exhibited better elution abilities towards the five NEOs than methylene chloride and ethyl acetate. Considering the polarity of NEOs, acetonitrile and methanol, which are strong polar solvents, were favorable for the desorption efficiency toward NEOs. Acetonitrile was used as the mobile phase for HPLC analysis and was selected as the preferred eluent for subsequent experiments to avoid solvent replacement.

#### 2.2.6. Effect of Elution Time

The desorption performance of the MSPE is associated with the desorption time. Herein, 6 mL of acetonitrile (6 mL) was used to optimize the elution time in the range of 2 to 10 min. As shown in Figure 3F, the recoveries of the five NEOs remained almost unchanged with longer desorption times, revealing that the NEOs were eluted in a shorter time. Hence, the optimal desorption time was 2 min.

### 2.3. Reusability of the Fe_3_O_4_@N-CMP

**Figure 4:**
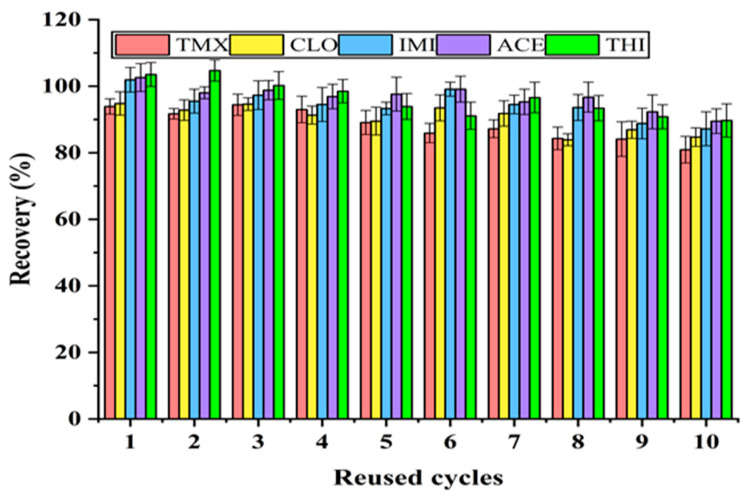
Reusability of Fe_3_O_4_@N-CMP on the extraction of five NEOs.

Reusability is a crucial parameter in MSPE assays, considering the lifespan and cost of the magnetic nanocomposites. The reusability of Fe_3_O_4_@N-CMP was assessed. Figure 4 shows that the adsorption capacity of Fe_3_O_4_@N-CMP is still maintained above 95% after 10 adsorption-regeneration cycles, proving the excellent reusability of Fe_3_O_4_@N-CMP. The MSPE approach based on Fe_3_O_4_@N-CMP was fitting for the analysis of NEOs because of its advantages, such as being rapid, easy, and having good material recoverability.

### 2.4. Possible Extraction Mechanism

The presence of many alkynyl groups and benzene rings in Fe_3_O_4_@N-CMP results in multiple π–π stacking interactions that play an essential role in the adsorption of NEOs. Moreover, electron-withdrawing groups in NEOs, such as nitro or cyano groups, endow NEOs with Lewis acidity. This facilitates Lewis acid-base interactions between NEOs and the benzene rings (as Lewis bases of Fe_3_O_4_@N-CMP) [50]. The negative electrostatic potential regions in NEOs, which can serve as nucleophilic sites, are concentrated in these nitro or cyano groups of the neonicotinoid. Electrostatic interactions between the nucleophilic sites of NEOs and quaternary ammonium groups in Fe_3_O_4_@N-CMP may enhance the adsorption efficiency towards NEOs [51]. Therefore, the proposed adsorption mechanism was ascribed to π–π stacking, Lewis acid-base, and electrostatic interactions.

### 2.5. Method Validation

To validate the MSPE-HPLC-DAD method based on Fe_3_O_4_@N-CMP, the linear range, limit of detection (LOD), limit of quantification (LOQ), and precision were determined under the optimal conditions described above. Ultrapure water was spiked with five NEOs to obtain concentrations in the range of 0.1 to 200 μg/L. This solution was utilized to determine linearity. Calibration curves were plotted using the areas of the chromatographic peaks measured at the spiked concentrations of each analyte. As presented in Table 1, good linearity was obtained in the range of 1.0 to 200 μg/L for ACE, and 1.5 to 200 µg/L for TMX, CLO, IMI, and THI, with the coefficient of determination (R^2^) ranging from 0.998 to 0.999. In accordance with the signal-to-noise ratios of 3 and 10, the LODs of the five NEOs ranged from 0.3 to 0.5 μg/L, and the LOQs ranged from 1.0 to 1.5 μg/L. Intra- and inter-day precisions, represented as RSDs, ranged from 2.2% to 4.2% and 1.4% to 3.6%, respectively. Five batches of Fe_3_O_4_@N-CMP were synthesized to evaluate reproducibility. The RSDs ranged from 3.7% to 6.0%, revealing the remarkable reproducibility of the fabrication of Fe_3_O_4_@N-CMP. The above results suggest that the established approach possesses good linearity, super-sensitivity, and outstanding precision, and can monitor NEOs in water samples.

### 2.6. Real Sample Analysis

**Figure 5:**
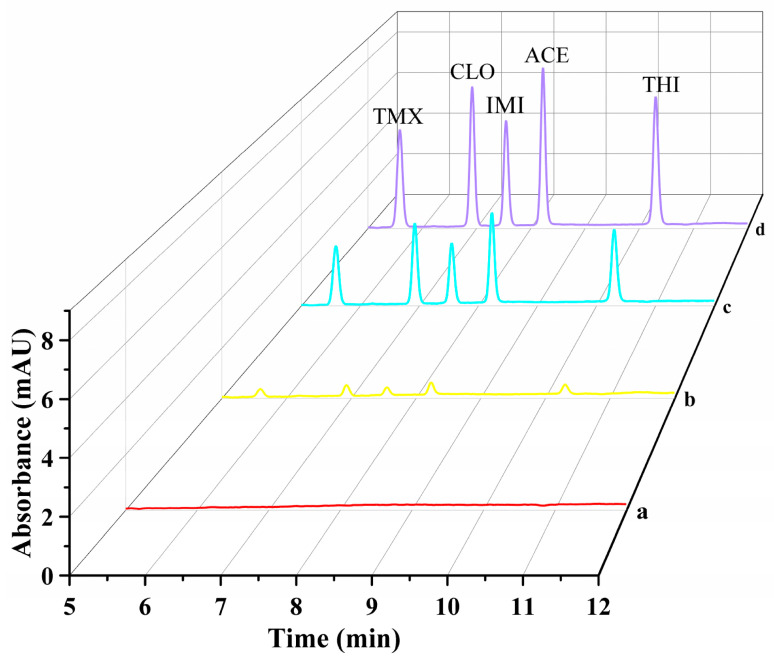
HPLC−DAD chromatograms of enriched NEOs from rainwater spiked with 0 (a), 5 (b), 50 (c), and 100 (d) μg/L, respectively.

Based on these positive results, the proposed MSPE-HPLC-DAD approach based on Fe_3_O_4_@N-CMP was used to quantify the levels of NEOs in tap water, rainwater, and lake water. As shown in Table 2, no NEO residues were detected in any of the three water samples. To appraise the accuracy of the current approach, recovery experiments were conducted using spiked water samples with NEO concentrations of 5, 50, and 100 μg/L. As shown in Table 2, the recoveries at the three spiked levels were 71.8–107.2% for tap water, 70.4–122.7% for rainwater, and 71.3–98.9% for Rainbow Lake water. The RSD values were less than 10%, confirming the reliability of our method. Typical chromatogram of the spiked NEOs in rainwater are shown in Figure 5. These results indicate that the established MSPE-HPLC-DAD method can be used to detect NEOs in environmental water samples.

### 2.7. Comparison with Other Methods

For a further evaluation of the analytical performance of the proposed method, it was compared with other methods reported in the literature (Table 3). Compared with SPME-UPLC-MS/MS [17], DSPE-UPLC-MS/MS [18,19], and MSPE-HPLC-MS [25,52], the current MSPE-HPLC-DAD method displayed higher LODs, because the MS detection promoted higher sensitivity towards NEOs than DAD detection. However, MS detection is more expensive than DAD detection. In addition, among the analytical methods using DAD [14,20], the TAPA-BPDA-COF-1-based SPE method exhibited lower LODs than the MSPE method; however, lesser amounts of adsorbents were used in the MSPE method. Moreover, among the MSPE methods, the established MSPE method possessed smaller adsorbent dosage, acceptable extraction time, and adequate LODs. Overall, the developed method is sensitive, economical, and practical, and can fulfill the demand for pre-concentration and determination of NEOs in environmental water samples.

## 3. Materials and Methods

### 3.1. Chemicals and Reagents

The five NEOs, including thiamethoxam (TMX), imidacloprid (IMI), acetamiprid (ACE), thiacloprid (THI), and clothianidin (CLO), were from Beijing Bailing Wei Technology Co., Ltd. (Beijing, China). The 1,3,5-triethynylbenzene (98% purity) and tetrakis(triphenylphosphine)palladium (Pd(PPh_3_)_4_; 99.4% purity) were from Bidepharm Technology Co., Ltd. (Shanghai, China). Anhydrous N, N-Dimethylformamide (DMF; 99.8% purity), triethylamine (Et_3_N; 99.5% purity), and copper(I) iodide (CuI; 98% purity) were from Energy Chemical Co., Ltd. (Huangshan, China). Methylene dichloride, methanol, acetonitrile (ACN), and ethyl acetate (all analytic grade) were purchased from Fuyu Fine Chemical Co., Ltd. (Tianjin, China). Other analytically pure chemicals Including sodium hydroxide and sodium chloride were obtained from Sinopharm Chemical Reagent Co., Ltd. (Shanghai, China). HPLC-grade acetonitrile (ACN) was purchased from Jianqiang Weiye Technology Co., Ltd. (Beijing, China). Hydrochloric acid (superior grade) was obtained from Far-East Fine Chemical Co., Ltd. (Yantai, China). Formic acid (FA) was obtained from Guangu Technology Co., Ltd. (Tianjin, China). Drinking water was from Wahaha Co., Ltd. (Jinan, China). Pure water was provided by the Ecology Institute of the Shandong Academy of Sciences, and the actual water samples were collected from tap water, rainwater and Rainbow Lake water (Jinan, China). The 1,4-dibromo-2,5-bis(bromomethyl)benzene and Fe_3_O_4_ were synthesized according to the reported procedures [53,54].

### 3.2. Instruments for Characterization

Scanning electron microscopy (SEM) was performed with a regulus 8100 SEM (Hi-tachi, Tokyo, Japan). Transmission electron microscopy (TEM) images were obtained with a JEM 2100 PLUS TEM (JEOL, Tokyo, Japan). Brunauer-Emmett-Teller (BET) surface areas were measured by a Micromeritics ASAP 2460 machine (Micromeritics Corporate, Norcross, GA, USA). Fourier-transform infrared (FT-IR) spectra were measured using a Nocolet Nexus 710 (Bruker, Rheinstetten, Germany). The composition and chemical and electronic states of the elements in the Fe_3_O_4_@N-CMP were measured using an Escalab 250 Xi X-ray photoelectron spectroscope (XPS) (Thermo Scientific, Waltham, MA, USA). Magnetization curves were calculated by a vibrating sample magnetometer (Mpms Squid Vsm, Quantum Design, San Diego, CA, USA).

### 3.3. Synthesis of Fe_3_O_4_@N-CMP

A 100 mL three-necked flask was charged with Fe_3_O_4_ nanospheres (100 mg), Pd(PPh_3_)_4_ (14 mg), CuI (4.6 mg), triethylamine (15 mL), and DMF (15 mL), followed by sonication for 15 min. Then, the mixture was mechanically stirred under 90 °C for 15 min under a nitrogen atmosphere. Then, 1,3,5-triethynylbenzene (30.1 mg) and 1,4-dibromo-2,5-bis(bromomethyl)benzene (126.5 mg) were added. After reacting at 90 °C for 24 h and cooling to room temperature, the obtained Fe_3_O_4_@N-CMP was collected by a magnet, washed three times with methanol and dichloromethane, and after being dried under vacuum at 60 °C overnight, 140 mg of Fe_3_O_4_@N-CMP was obtained.

### 3.4. Preparation of Mixed Standard NEOs Solutions and Actual Samples

A mixed standard solution with a concentration of 50.0 ug/mL and a volume of 100 mL was prepared from 5 mg of each of the five NEOs (TMX, IMI, ACE, THI, and CLO) in purified water and stored in a refrigerator at 4 °C. The working solution was obtained by diluting the mixed standard solution with purified water. In this work, three types of surface water, including tap water (Jinan, China), rainwater from 23 September 2023 (Jinan, China), and Rainbow Lake water from 28 September 2023 (Jinan, China) were selected as real environmental samples. The water samples were first stored in a refrigerator at 4 °C and then analyzed after filtration through a 0.22 µm microporous filtration membrane.

### 3.5. MSPE Procedure

First, 10 mg of Fe_3_O_4_@N-CMP was added to an EPA sample bottle, and then 30 mL of the sample solution (25 µg/L) was added to the sample bottle. The mixture was shaken in a constant temperature oscillator 250 times per minute for 20 min at room temperature. Then, the Fe_3_O_4_@N-CMP was collected from the water phase by using an external magnet on the outside of the sample vial, and the supernatant was also discarded. The adsorbed target analytes were released from the Fe_3_O_4_@N-CMP by HPLC-ACN under ultrasonic shaking for 6 min, and the operation was repeated. The eluted solution was dried at 45 °C under N_2_. Finally, the residue was re-dissolved in 1 mL of HPLC-ACN, and these resolubilized samples were filtered through a 0.45 µm white organic membrane and then injected into HPLC-DAD for analysis. In addition, the adsorbent can be recycled. The MSPE procedure is shown in Scheme 1.

### 3.6. HPLC-DAD Determination

The HPLC experiments were carried out on an Agilent Technologies 1260 Infinity II liquid chromatography system consisting of an autosampler, a quaternary pump (Agilent Technologies, Santa Clara, CA, USA), a thermo-static column chamber, a DAD detector, and a workstation to deal with the chromatographic data. A Symmetry-C18 column (250 × 4.6 mm, 5 µm) was used for sample separation at room temperature. The injection loop volume was 5 µL. Chromatographic separation was performed with a mobile phase consisting of HPLC acetonitrile (C) and water containing 0.1% FA (D) at a flow rate at 1 mL/min. The UV monitoring wavelength was set at 257 nm. The operation was conducted in a sequential mode for a total time of 24 min, with the first 12 min being the time for single sample analysis, and the last 12 min being the equilibrium column pressure time. The gradient elution conditions were as follows: 0–1 min for 25% acetonitrile, 1–5 min for 25–35% acetonitrile, 5–12 min for 35–45% acetonitrile, 12–15 min for 45–25% acetonitrile, and 15–24 min for 25% acetonitrile. The five NEOs were separated with the elution order of TMX, CLO, IMI, ACE, and THI.

## 4. Conclusions

Fe_3_O_4_@N-CMP containing quaternary ammonium groups was successfully constructed and utilized as a reusable MSPE adsorbent toward NEOs in real water samples. The Fe_3_O_4_@N-CMP exhibited satisfactory adsorption performance for NEOs by virtue of the synergistic effects of π–π stacking interactions, Lewis acid-base interactions, and electrostatic interactions. In combination with HPLC-DAD, the proposed approach achieved a wide linearity, low LODs/LOQs, excellent precision, and acceptable reproducibility. Finally, the Fe_3_O_4_@N-CMP-MSPE-HPLC-DAD method was successfully applied to the sensitive detection of NEOs in tap water, rainwater, and lake water. Adequate recoveries were obtained in the range of 70.4% to 122.7%. These results indicate that using Fe_3_O_4_@N-CMP as a MSPE sorbent can offer an accurate, practical, and reproducible method to determine NEOs present in environmental water samples.

## Figures and Tables

**Table 1:** Analytical performances of Fe_3_O_4_@N-CMP for HPLC-DAD determination of NEOs.

Analytes	Linear Range (μg/L)	R^2^	LODs (μg/L)	LOQs (μg/L)	RSDs (%)		
					Repeatability (RSD%, n = 5) Inter-Day	Repeatability (RSD%, n = 5) Intra-Day	Repeatability (RSD%, n = 5) Batch to Batch
TMX	1.5–200	0.998	0.5	1.5	2.5	4.2	6.0
CLO	1.5–200	0.999	0.5	1.5	1.4	3.7	5.6
IMI	1.5–200	0.999	0.5	1.5	1.5	2.2	6.0
ACE	1.0–200	0.999	0.3	1.0	3.0	3.1	3.7
THI	1.5–200	0.999	0.5	1.5	3.6	2.7	3.8

**Table 2:** Analytical results of NEOs in real water samples.

Analytes	Linear Range (μg/L)	RSDs (%)				
		TMX	CLO	IMI	ACE	THI
Tap water	0	ND	ND	ND	ND	ND
	5	89.9 ± 6.5	86.2 ± 3.1	94.2 ± 4.4	96.5 ± 1.3	107.2 ± 3.7
	50	80.4 ± 7.1	87.0 ± 6.0	91.5 + 5.6	94.4 ± 5.4	96.9 ± 6.5
	100	71.8 ± 1.6	78.4 ± 1.9	84.4 ± 1.6	86.8 ± 3.1	90.7 ± 2.5
Rain water	0	ND	ND	ND	ND	ND
	5	93.1 ± 4.2	104.8 ± 4.4	106.3 ± 2.3	110.0 ± 0.9	122.7 ± 4.2
	50	70.4 ± 1.0	79.7 ± 0.6	83.1 ± 1.1	85.1 ± 0.4	87.3 ± 0.1
	100	73.1 ± 2.6	77.5 ± 3.2	81.2 ± 2.6	83.6 ± 2.3	87.2 ± 1.8
Lake water	0	ND	ND	ND	ND	ND
	5	79.9 ± 1.0	92.0 ± 1.0	98.6 ± 3.5	98.9 ± 3.2	94.8 ± 0.5
	50	71.3 ± 2.5	82.0 ± 2.2	88.6 ± 1.5	88.8 ± 2.1	94.6 ± 0.3
	100	72.9 ± 5.9	78.0 ± 3.1	84.8 ± 2.5	85.3 ± 2.6	91.4 ± 0.5

ND: not detected.

**Table 3:** Comparison with other analytical methods for the determination of neonicotinoids.

Matrix	Method	Analytes	Adsorbent	Dosage (mg)	Extraction Time (t)	LODs (μg/L)	Ref.
Environmental water	MSPE-HPLC-UV	TMX, IMI, ACE, THI	MOPC-ZSM-5	10	20	0.1–0.2	[20]
Environmental water	MSPE-HPLC-MS	IMI, ACE, THI	MOF-199/Fe_3_O_4_	50	20	0.3–1.5	[52]
Environmental water	MSPE-HPLC-MS	TMX, CLO, IMI, ACE, THI	Fe_3_O_4_/GO/ZIF-67	40	50	0.06–1.0	[25]
Lettuce	SPME-UPLC-MS/MS	TMX, CLO, IMI, ACE, THI, NIT, DIN	The water-swelling fiber	-	10	0.03–0.11	[17]
Fruit juices and tea beverages	DSPE-UPLC-MS/MS	IMI, ACE, THI	MIL-101(Cr)	20	5	0.0019–0.02	[19]
Lake water	SPE-HPLC-DAD	IMI, ACE, THI, TMX	TAPA-BPDA-COF-1	30	25	0.08–0.12	[14]
Medicine and food homology products	DSPE-UPLC-MS/MS	IMI, ACE, THI, NIT, DIN	MWCNTs/NH_2_-MIL-101(Fe)	30	6	0.01–0.07 μg/kg	[18]
Environmental water	MSPE-HPLC-DAD	TMX, CLO, IMI, ACE, THI	Fe_3_O_4_@N-CMP	10	20	0.3–0.5	This work

## Data Availability

Data is contained within the article.
